# Golden Hour perception in a public maternity hospital: challenges and potentialities under a multi-professional team’s vision

**DOI:** 10.1590/1984-0462/2025/43/2025158

**Published:** 2025-12-01

**Authors:** Juliana Rodrigues Dias, Cristina Ortiz Sobrinho Valete

**Affiliations:** a Santa Casa de Misericórdia de São Carlos, São Carlos, SP, Brazil.; b Universidade Federal de São Carlos, São Carlos, SP, Brazil.

**Keywords:** Umbilical cord clamping, Breastfeeding, Humanizing delivery, Clampeamento do cordão umbilical, Aleitamento materno, Humanização do nascimento

## Abstract

**Objective:**

The aim of this study was to investigate the self-perception of health professionals regarding the difficulties in the implementation of breastfeeding, skin-to-skin contact, and delayed cord clamping in the delivery room, and how difficulties and practices are related.

**Methods:**

This is an observational, cross-sectional, and quantitative study conducted with health professionals who work in the delivery room in a public maternity hospital in the state of São Paulo. The questionnaire was self-answered in January 2025. The epidemiological characteristics of health professionals, and questions related to breastfeeding, skin-to-skin contact, and delayed cord clamping in the delivery room were analyzed. Descriptive statistics were performed. The cutoff to consider an item as a difficulty was an agreement below 50%. Difficulties with breastfeeding, skin-to-skin contact, and delayed cord clamping were analyzed through multiple correspondence analyses.

**Results:**

A total of 75 health professionals were included. The lack of health professionals (38%), inadequate physical space (36%), and low adherence of obstetricians (34%) were considered difficulties. Correspondence analysis revealed that difficulties did not correspond to practices. Inadequate physical space and a lack of health professionals were correlated. When doctors indicate breastfeeding, they indicate delayed cord clamping, suggesting that these practices go hand in hand.

**Conclusions:**

The findings suggest a lack of alignment among health professionals. Insufficient numbers of health professionals, physical limitations, and non-adherence of obstetricians are barriers to overcome. Breastfeeding and delayed cord clamping appear to be integrated practices. Other factors may influence these practices, as difficulties did not correspond with them.

## RESUMO

**Objetivo:**

Investigar a autopercepção de profissionais de saúde quanto às dificuldades para a implementação da amamentação, contato pele a pele e clampeamento tardio do cordão na sala de parto, e como as dificuldades e as práticas estão relacionadas.

**Métodos:**

Estudo observacional, transversal e quantitativo, com profissionais de saúde que atuam em sala de parto em uma maternidade pública do estado de São Paulo. O questionário foi autorrespondido em janeiro de 2025. Foram analisadas as características dos profissionais de saúde e os aspectos relacionados à amamentação, contato pele a pele e clampeamento tardio de cordão na sala de parto. Foi realizada análise descritiva. O ponto de corte para considerar um item como dificuldade foi a concordância inferior a 50%. As dificuldades e as práticas foram avaliadas por análise de correspondência múltipla.

**Resultados:**

Foram incluídos 75 profissionais. Entre as dificuldades observadas, destacam-se a falta de profissionais de saúde (38,6%), o espaço físico inadequado (36,0%) e a baixa aderência dos obstetras às práticas (34,6%). A análise de correspondência revelou que as dificuldades não corresponderam às práticas. O espaço físico inadequado e a falta de profissionais apresentaram correspondência. Quando médicos indicam amamentação, indicam o clampeamento tardio do cordão, sugerindo que essas práticas andam juntas.

**Conclusões:**

Os achados sugerem que haja falta de alinhamento entre os profissionais. A falta de profissionais, as limitações de espaço e a não aderência de obstetras são barreiras a serem vencidas. Para os profissionais, a amamentação e o clampeamento oportuno do cordão são práticas que caminham juntas. Outros fatores parecem influenciar essas práticas, uma vez que elas não corresponderam com as dificuldades encontradas.

## INTRODUCTION

Infant mortality rates have decreased over the years. However, neonatal mortality remains responsible for an important part of childhood deaths.^1^ Of the 4.9 million deaths of children under 5 years in 2022, 2.3 million occurred during the first month of life.^2^ Decreasing preventable infant deaths requires targeted investments in quality, available, and affordable healthcare, including skilled healthcare personnel at childbirth, prenatal and postnatal care, neonatal care, preventative services such as vaccination, and diagnostic, preventive, and curative measures to treat the leading causes of infant death.^2^

The first hour of a neonate’s life is known as the Golden Hour (GH).^3^ During this period, practices should be carried out, which should be started immediately after birth as long as the mother and the neonate are in good clinical condition. Delayed cord clamping, skin-to-skin contact, and breastfeeding during the first hour are part of this strategy.^4,5^ The GH, when carried out through institutional protocols, promotes higher breastfeeding levels, reducing maternal and neonatal mortality and increasing the mother-baby bonding.^6^

Delayed cord clamping, for a minimum of 60 s, facilitates cardiorespiratory transition during labor, improves hematological parameters in the neonatal period and iron stores during infancy, making it an important public health strategy for preventing iron deficiency anemia, which is still a global health problem.^7^ Still, skin-to-skin contact consists of placing the neonate in direct contact with the mother’s lap, in the abdomen, or chest region. This practice promotes control of the neonate’s body temperature, cardiorespiratory stability, and reduces the risk of hypoglycemia, in addition to favoring the first feeding. For the mother, this contact favors the establishment of bonds, reduction of stress, and reduction of the risk of postpartum hemorrhage.^8^ Early sucking stimulates the pituitary gland to produce prolactin and oxytocin, increasing the body’s milk production and having effects on faster uterine involution and less bleeding.^9^ Also, breastfeeding during the GH is step 4 of the Baby-Friendly Hospital Initiative.^10^

Although the literature highlights the various benefits of these practices, they are still not routine in all healthcare institutions.^11^ There are obstacles to adopting these practices, such as local conditions, medical practices, lack of time, reduced number of professionals, and lack of adequate team training.^12^ Even neonates with good vitality often undergo immediate interventions that could be postponed or readapted, such as drying in a heated crib, airway aspiration, ocular prophylaxis, and other procedures, to the detriment of humanized care.^3^ Routine exams and procedures should only be performed after establishing skin-to-skin contact, except in cases of medical indication.^13^ Considering this, studies that analyze local difficulties in the implementation of these practices are welcome, as they can help understand how they can be improved.

This study aims to investigate the self-perception of health professionals regarding the difficulties in the implementation of breastfeeding, skin-to-skin contact, and delayed cord clamping in the delivery room, and how difficulties and practices are related.

## METHOD

This observational and cross-sectional study was conducted in a public maternity hospital located in the state of São Paulo. The inclusion criteria were health professionals who worked in the delivery room. The exclusion criteria were professionals on leave or vacation during the study period. This study followed Resolution 196/96 from the National Health Council and was approved by the institutional Ethics and Research Committee. All participants signed the informed consent.

Data were collected in January 2025 through a REDCap (Research Electronic Data Capture) questionnaire, which was administered in person during a work shift. The sample size was calculated using Raosoft (www.raosoft.com), considering a 50% difference between responses, a 5% error, and a 95% confidence interval, which resulted in a minimum of 72 participants. The self-answered questionnaire investigated health professionals’ epidemiological characteristics (age, sex, area of activity, years of experience in the delivery room, and highest level of training in the area), in addition to the domains of knowledge (six questions), infrastructure (three questions), medical team (five questions), neonate’s conditions (three questions), and maternal conditions (four questions). Answers were organized on a five-point Likert scale (1 - completely disagree to 5 - completely agree). Two open questions: “What do you consider to be hindering factors in carrying out GH in the delivery room?” and “In your opinion, what would make it easier to practice GH in the delivery room?”

Statistical analyses were performed in three steps. First, health professionals’ characteristics were identified. Then, difficulties were defined considering agreement in the questionnaire’s items. Finally, the difficulties were analyzed together with practices (indicating breastfeeding, skin-to-skin contact, and delayed cord clamping). Descriptive statistics were performed with the calculation of frequencies, medians, and interquartile ranges (IQR). The two open questions were analyzed using a word cloud created in a document on the Google platform, which identified the most central and largest words as the most relevant. The reliability of the questionnaire answers was assessed by calculating Cronbach’s alpha. The answers “I agree with” and “I completely agree with” were grouped as agreement, and the others were grouped as non-agreement. Questions with unfavorable answers, considered those with less than 50% agreement, were considered as difficulties. Difficulties were analyzed with doctors indicating breastfeeding, skin-to-skin contact, and delayed cord clamping, using multiple correspondence analysis. Multiple correspondence analysis is a descriptive technique that graphically represents the intersection between more than two categorized variables through the symmetrical map of correspondences. The proximity of plotted points suggests that there is correspondence.^14^ All analyses were performed using Stata version 18.0 (Stata Corp, LC). The writing of this article followed the recommendations of the STROBE form for observational studies.^15^

## RESULTS

The sample comprised 75 health professionals. Many were female (92%), 57% were physicians, and 49% had between 2 and 10 years of experience in the delivery room (Table 1).

**Table 1:** Health professionals’ characteristics.

Variable	
Age in years (median /IQR)	32 (28-37)
Female sex (n/%)	69 (92)
Area of activity	
Nurse (n/%)	11 (15)
Nursing assistant (n/%)	5 (7)
Nursing technician (n/%)	16 (21)
Physician (n/%)	43 (57)
Years of experience in the delivery room (n/%)	
<1	16 (21)
1 to 2	13 (17)
2 to 10	37 (50)
>10	9 (12)
The highest level of training in the area (n/%)	
Technician	21 (28)
Under graduation	34 (45)
Post-graduation *lato sensu*	16 (21)
Post-graduation *stricto sensu*	4 (6)

IQR: Interquartile range.

The word cloud analysis of the question “What do you consider to be hindering factors in carrying out GH in the delivery room?” resulted in aspects related to the team, “lack”, and “rush” as relevant. On the other hand, training was considered a relevant facilitator.

Cronbach’s alpha was 0.7130. Regarding knowledge, it was observed that, about having been trained on GH in the last year, 50% of health professionals said they were trained, and 98% said they think that GH is important. Concerning the infrastructure and medical team, 38% agree that the number of health professionals is adequate. Thirty-six agree that the physical space is adequate. Also, 34% agree that obstetricians generally request GH. About maternal and neonatal conditions, 77% of health professionals agree that, rarely, the neonate’s clinical conditions do not allow skin-to-skin contact (Table 2).

**Table 2:** Health professionals’ perceptions regarding the Golden Hour.

Variable	Agree (n/%)
Do you know the institutional GH protocol?	70 (93)
Do you think GH interventions are important?	74 (98)
Have you received any training about GH in the last year?	38 (50)
When the neonate does not need resuscitation, can the umbilical cord be clamped after 1–3 min?	71 (94)
Immediately after birth, should the neonate be put on skin-to-skin contact during the first hour?	70 (93)
Doctors generally recommend breastfeeding within the first hour	67 (89)
Doctors usually recommend skin-to-skin contact within the first hour	54 (72)
Doctors often recommend delayed cord clamping	57 (76)
Obstetricians generally request GH to be performed	26 (34)
Pediatricians generally request GH to be performed	50 (66)
Is the room temperature appropriate?	40 (53)
Is the number of health professionals adequate?	29 (38)
Is the physical space of the delivery room adequate for skin-to-skin contact and breastfeeding in the first hour? n (%)	27 (36)
Rarely, the clinical conditions of the neonate do not allow for delayed cord clamping	49 (65)
Rarely, the clinical conditions of the neonate do not allow skin-to-skin in the first hour of life	58 (77)
Rarely, the clinical conditions of the neonate do not allow breastfeeding in the first hour of life	50 (66)
Cesarean section should not prevent GH	64 (85)
The mother’s sedation does not interfere with GH. Mothers are not over-sedated	43 (57)
Mothers are tested for HIV; this is done at the hospital, and does not interfere with GH	50 (66)
Do you think women want to put their babies in skin-to-skin contact during the first hour?	53 (70)

HIV: Human immunodeficiency virus; GH: Golden Hour.

**Figure 1:**
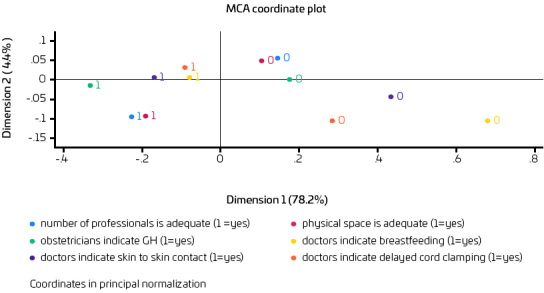
Multiple correspondence analysis of difficulties and doctors’ indications for breastfeeding, skin-to-skin contact, and delayed cord clamping.

The multiple correspondence analysis between the difficulties and practices indicates that when doctors indicate breastfeeding, they also tend to indicate delayed cord clamping. This can be observed by the proximity between the colored points that represent these answers. Professionals who think that the physical space is adequate also think that the number of health professionals is adequate. The reported difficulties did not correspond with the practices analyzed (Figure 1).

MCA: multiple correspondence analysis.

## DISCUSSION

In this study, difficulties in implementing GH were identified through health professionals’ perceptions. The number of health professionals, the physical space, and the obstetricians requesting GH were reported. The team’s hurry was associated with difficulties in implementing GH, and training was recognized as a facilitator. In their opinion, when doctors indicate breastfeeding, they indicate delayed cord clamping. Practices and difficulties did not correspond.

It is essential that measures are adopted in all maternity wards to prioritize skin-to-skin contact, delayed cord clamping, and breastfeeding in the first hour of life, and that only necessary interventions are performed during and after delivery.^16^ The importance of these practices, according to recommendations from the WHO and the Baby-Friendly Hospital Initiative, has been well documented.^17^ Knowledge of the factors involved in adopting these measures, therefore, is essential.

Some issues highlighted in the present study indicate that team dynamics can negatively impact practices, and this factor should be considered. Therefore, it is crucial to address team structure and time management to create a more balanced and productive environment. This was also reported in the word cloud results, as “lack” and “rush” were relevant. Also, regarding the medical team, low agreement was observed among obstetricians requesting GH, which constitutes another very important point to be considered. This lack of alignment among health professionals can result in inconsistent implementation of protocols, directly impacting their experience, the experience of mothers, and the neonates’ experiences.^17^ Obstetricians’ hesitation in requesting the GH may be related to a myriad of factors, such as a lack of knowledge about the benefits of this practice, a lack of effective communication among team members, or the perception that this practice is the exclusive responsibility of the pediatrician. Lack of organization and absence of defined duties were also reported barriers in a Brazilian maternity.^18^ In the “Nascer no Brasil” study, which refers to the years 2016–2017, it was observed that, in births assisted by nurse-midwives, breastfeeding rates in the first hour were higher when compared to the rates of births assisted by obstetricians, suggesting better practices for nurse-midwives.^19^

It is important to emphasize that all health professionals working in the delivery room have an important role in promoting early breastfeeding, including obstetricians. Nurses can encourage other team members involved in childbirth care, promoting awareness, information exchange, and support for breastfeeding in the first hour of life. To achieve this goal, there must be effective communication among the team.^20^ Training that highlights not only the clinical benefits but also the relevance and emotional impact of these practices can be fundamental in changing this perception. Furthermore, establishing an open dialogue between obstetricians and pediatricians to foster a unified team approach can facilitate the creation of integrated protocols, ensuring that all health professionals involved in childbirth care have the same goals and objectives. In this way, greater commitment from the medical team to requesting skin-to-skin contact, breastfeeding, and delayed cord clamping will significantly contribute to the effectiveness of interventions, promoting a more collaborative environment focused on improving the quality of neonatal care.^20^

Concerning the infrastructure, the number of health professionals, and the physical space were reported to interfere with the implementation of the practices. This result agrees with other studies. Silva et al., in Vitória, observed that physical structure and lack of human resources were barriers to a GH protocol implementation.^18^ Work overload can lead to staff stress, hindering full attention to better practices.^21^ Thus, investing in adequate infrastructure and a sufficient number of qualified health professionals not only improves patients’ experience but also contributes to their satisfaction and the efficiency of care. The literature emphasizes that prepared environments and sufficient teams are determining factors in improving the quality indicators of neonatal care, reinforcing the importance of prioritizing these aspects in health policies and institutional care processes.^22^ This way, adequate infrastructure seems to be essential in the health professionals’ opinion.

Regarding training on the GH protocol, almost half of the health professionals agreed they had received it in the last year. Silva et al. emphasized that health professionals reported that protocols are important and do not require many investments to be implemented. In that study, they also reported that protocols need to be adapted to local reality, and training was the most important facilitator.^18^ Training is essential to promote better practices. To transform the care model, which is still heavily interventionist, it is necessary to make significant changes and break paradigms. This process must be continuous, dynamic, and integrated, demanding a well-trained and committed multidisciplinary team aligned with institutional principles and established policies.^8^ This result points out that training was considered important in this scenario.

Maternal and neonatal conditions do not appear to have interfered with the health professionals’ perceptions about the GH. Although the literature suggests that factors such as the mother’s general health and complications during delivery can impact the neonate’s experience,^6^ this does not appear to be a problem in the evaluated scenario. However, it is worth noting that attention to maternal conditions is still crucial, as other aspects of care, such as emotional and physical support, can influence the overall experience of the mother and neonate. According to the literature, the neonate’s clinical condition is a relevant factor for the performance of skin-to-skin contact and breastfeeding in the first hour of life.^3^ It is known that preterm birth increases the likelihood of interventions to ensure clinical stability, which, in turn, reduces the opportunities for delayed cord clamping, skin-to-skin contact, and breastfeeding in the first hour of life. A study conducted by Sousa et al. shows that the Apgar score at the first minute is a good predictor of breastfeeding in the first hour of life, since the neonate’s vitality is directly related to the need for immediate care, which can hinder immediate contact with the mother.^23^

In the present study, the multiple correspondence analysis showed that in the health professionals’ opinion, breastfeeding and delayed cord clamping go hand in hand, indicating that these practices seem to be promoted together in the form of integrated care; that is, when one intervention is recommended, the other is also. Skin-to-skin contact was not as close as the other practices, suggesting a local fragility. This fragility was already reported in the literature. Furthermore, the inadequate physical space and the lack of health professionals were correspondent but did not reveal correspondence with the recommended practices, suggesting that, although health professionals report those difficulties, they do not go hand in hand with practices. Other factors not explored in the present study could explain this result, which expresses health professionals’ opinions. In Brazil, a study revealed that breastfeeding was more frequent than skin-to-skin contact.^23^ In Nepal, skin-to-skin contact alone was the least performed practice, occurring in only 19.5% of the births analyzed, despite the impact that this practice has on reducing neonatal mortality. Breastfeeding within the first hour was observed in 30.1% of cases, and timely cord clamping in 68.1%. Different from the present study, the authors observed these practices directly.^24^ Although we did not measure skin-to-skin contact directly, these results suggest that a fragility for skin-to-skin contact may exist in the maternity studied. This was already pointed out in the discussion. Most of all, if difficulties did not present correspondence with practices, future studies should investigate other factors, quantitatively and qualitatively, using mixed methods, to explain why these practices are not systematically adopted by health professionals.

Although health professionals know the institutional protocols, they think that the GH is important, and agree that delayed cord clamping, skin-to-skin contact, and breastfeeding should be done; these results do not guarantee that these practices are implemented in practice. Again, we did not measure these practices directly. We measured health professionals’ perceptions about them, which is an important issue, and they think that not all pediatricians request these practices, and few obstetricians do this. Literature emphasizes that there is a gap between knowledge and practice that needs to be addressed. Considering this, proper conditions should be given to translate this knowledge into better practices.^25,26^ The biomedical model of care limits a transdisciplinary approach and the achievement of better practices. To help reduce this gap, quality improvement strategies could bring care into standardized protocols with well-defined papers for each health professional and a more horizontal relationship.^27,28^

This study has limitations. It analyzed the self-perception of health professionals regarding GH and did not directly measure adherence to the GH protocol, which prevents concluding the actual implementation of GH in practice. Another important point is that only a single facility was studied, which limits extrapolations to other services, especially those with different clinical profiles. On the other hand, this study adds to the body of evidence on GH, offering specific contributions regarding the self-perception of health professionals, which should be valued for GH to be effectively implemented.

In conclusion, in the scenario analyzed, there is a lack of alignment among health professionals and a need for structural improvements. The lack of health professionals, physical limitations, and non-adherence of obstetricians are barriers to overcome. In the health professionals’ opinion, breastfeeding and delayed cord clamping appear to be integrated practices. Other factors may influence these practices, as difficulties did not correspond with them, suggesting that the implementation of the GH does not rely only on guidelines and protocols but on a collective commitment from the multidisciplinary team.

## Data Availability

The database that originated the article is available with the corresponding author.
